# Human Mitotic Centromere-Associated Kinesin Is Targeted by MicroRNA 485-5p/181c and Prognosticates Poor Survivability of Breast Cancer

**DOI:** 10.1155/2019/2316237

**Published:** 2019-04-03

**Authors:** Huajun Lu, Chaoqun Wang, Lijun Xue, Qi Zhang, Frank Luh, Jianghai Wang, Tiffany G. Lin, Yun Yen, Xiyong Liu

**Affiliations:** ^1^Department of Oncological Radiotherapy, Affiliated Dongyang People's Hospital of Wenzhou Medical University, Dongyang, Zhejiang 322100, China; ^2^Department of Pathology, Affiliated Dongyang People's Hospital of Wenzhou Medical University, Dongyang, Zhejiang 322100, China; ^3^Department of Pathology, Loma Linda University Medical Center, Loma Linda, CA 92354, USA; ^4^Department of Bioinformatics, Hangzhou Hepu Biotechnology Inc., Hangzhou, Zhejiang 310015, China; ^5^Sino-American Cancer Foundation, Temple City, CA 91780, USA; ^6^Department of Tumor Biomarker Development, California Cancer Institute, Temple City, CA 91780, USA

## Abstract

**Purpose:**

This study aims to evaluate the prognostic value of human Mitotic Centromere-Associated Kinesin (MCAK), a microtubule-dependent molecular motor, in breast cancers. The posttranscriptional regulation of MCAK by microRNAs will also be explored.

**Methods:**

The large-scale gene expression datasets of breast cancer (total n=4,677) were obtained from GEO, NKI, and TCGA database. Kaplan-Meier and Cox analyses were used for survival analysis. MicroRNAs targeting MCAK were predicted by bioinformatic analysis and validated by a dual-luciferase reporter assay.

**Results:**

The expression of MCAK was significantly associated with aggressive features of breast cancer, including tumor stage, Elston grade, and molecular subtypes, for global gene expression datasets of breast cancer (p<0.05). Overexpression of MCAK was significantly associated with poor outcome in a dose-dependent manner for either ER-positive or ER-negative breast cancer. Evidence from bioinformatic prediction, coexpression assays, and gene set enrichment analyses suggested that miR-485-5p and miR-181c might target MCAK and suppress its expression. A 3'UTR dual-luciferase target reporter assay demonstrated that miR-485-5p and miR-181c mimics specifically inhibited relative Firefly/Renilla luciferase activity by about 50% in corresponding reporter plasmids. Further survival analysis also revealed that miR-485-5p (HR=0.59, 95% CI 0.37-0.92) and miR-181c (HR=0.54, 95% CI 0.34-0.84) played opposite roles of MCAK (HR=2.80, 95% CI 1.77-4.57) and were significantly associated with better outcome in breast cancers.

**Conclusion:**

MCAK could serve as a prognostic biomarker for breast cancers. miR-485-5p and miR-181c could specifically target and suppress the MCAK gene expression in breast cancer cells.

## 1. Background

Microtubules (MTs) are essential biological polymers of fundamental importance for mitosis in eukaryotic cells. The human Mitotic Centromere-Associated Kinesin (MCAK) gene, also recognized as Kinesin Family Member 2C (KIF2C), encodes a kinesin-like protein that can depolymerize microtubules at the plus end, thereby promoting mitotic chromosome segregation during mitosis [1]. MCAK can interact with KIF18B to form an MCAK-KIF18B complex, which is negatively regulated by Aurora kinases through phosphorylation of MCAK [2]. Aurora kinases regulate MT plus-end stability through control of MCAK-KIF18B complex formation to constitute the major microtubule plus-end depolymerizing activity in mitotic cells. MCAK and KIF2B stimulate kinetochore-microtubule dynamics during distinct phases of mitosis to correct malorientations [3]. MCAK plays a role in chromosome congression and is required for the lateral to the end-on conversion of the chromosome-microtubule attachment [4]. Both protein and mRNA levels of MCAK were upregulated in colorectal cancer, and expression levels correlated strongly with Ki-67 expression [5]. Overexpression of MCAK was also considered an independent predictor of overall survival and lymph node metastasis in colorectal cancer [6]. The MCAK gene expression was also found to be increased in glioma samples and associated with histopathological grades that impact poor survival of glioma [7].

Breast cancer is a common malignant disease among women in the world [8, 9]. Because of the heterogeneity of breast cancer cells, there is tremendous variation in clinical outcomes [10, 11]. Molecular-based classification of breast cancers has been widely used to predict outcomes and select the appropriate therapeutic regimen for patients. Currently, more therapeutic targets and corresponding inhibitors for breast cancers are being explored to improve treatment efficacy with fewer adverse side effects. Here, we hypothesize that MCAK could be a driver gene for tumorigenesis and could serve as prognostic biomarkers and/or therapeutic targets for breast cancer treatment.

In many cases, microRNAs play essential roles in gene regulation [12]. miR-485-5p has been reported to suppress mitochondrial respiration, cell migration, and invasion in breast cancer cell lines [13]. In oral tongue squamous cells, miR-485-5p antagonizes PAK1 to reverse epithelial to mesenchymal transition and promote cisplatin-induced cell death [14]. miR-485-5p also could serve as a prognostic biomarker and associate with better survival in gastric cancer [15–17]. Other microRNAs like miR-181c were reported to reduce the proliferation, migration, and invasion of neuroblastoma cells through targeting Smad7 [18]. However, another report demonstrated that miR-181c functioned as an oncogene and promoted proliferation through inhibiting PTEN protein expression by targeting 3'-UTR of PTEN mRNA in inflammatory breast cancer SUM149 cells [19]. The mature form of miR-181c could also translocate into mitochondria and suppress the mitochondrial function through targeting of the mt-Cox1 gene [20]. Moreover, miR-181c was also reported to be involved in chemoresistance and antagonized long non-coding RNA GAS5 in pancreatic cancers [21, 22]. It also contributed to the resistance of cisplatin in non-small cell lung cancer cells by targeting Wnt inhibition factor 1 [23]. Neither miR-485-5p nor miR-181c has been previously reported to target MCAK gene and reduce its expression level in cancers.

Here, we explored the clinical meaning and prognostic significance of MCAK by using 13 independent breast cancer datasets from Gene Expression Omnibus (GEO) and the Cancer Genome Atlas (TCGA). All eligible microRNAs that target MCAK were predicted by using bioinformatics and biostatistics analysis and validated by dual-luciferase 3'-UTR report assay. The clinical significance of MCAK and above two microRNAs were also observed.

## 2. Materials and Methods

### 2.1. Breast Cancer Cell Culture

MCF-7 (ER-positive) and MDA-MB-231 (ER-negative) cell lines were obtained from ATCC (American Type Culture Collection, Manassas, VA USA) in June 2011 and September 2013. Cells were incubated with 5% CO_2_ at 37°C in a humidified incubator in Dulbecco's Modification of Eagle's Medium (DMEM) (Mediatech, Inc., Manassas, VA, USA) supplemented with 10% fetal bovine serum (FBS) (Omega Scientific, Inc., Tarzana, CA, USA) and penicillin and streptomycin (Thermo Fisher Scientific Inc.). Frozen aliquots were stored in liquid nitrogen vapor phase when we obtained cells from ATCC for long-term storage. Cells were cultured for no longer than six months after thawing. Cell lines were authenticated by ATCC before delivery and not reauthenticated in our laboratory.

### 2.2. pmirGLO Dual-Luciferase miRNA Target Reporter Assay

The pmirGLO dual-luciferase miRNA target expression vectors (Promega) were constructed as reporter plasmids. miR-485-5p and miR-181c, which target MCAK sense/antisense oligonucleotides, were annealed and then inserted into multiple cloning sites (MCS, PmeI, and XbaI) in the 3' untranslated region (UTR) of Firefly (luc2) gene in the pmirGLO vector.

About 5-10×10^5^ MCF7 cells were seeded in each well of a 6-well plate and incubated at 37°C with 5% CO_2_ overnight. The human miR-485-5p and miR-181c mimics were obtained from Vigene Biosciences (Rockville, MD). These pmirGLO reporter vectors and miRNA mimics were transfected in antibiotic-free Opti-MEM medium (Life Technologies, Carlsbad, CA, USA) with Lipofectamine 3000 reagent (Life Technologies) according to the manufacturer's instructions. Luciferase activity was performed 48 hours after transfection.

### 2.3. Dual-Luciferase Determination

Cells were plated into 24-well plates and transfected with pmirGLO-485-WT, pmirGLO-485-Mut, pmirGLO-181c-WT, or pmirGLO-181c-Mut, with corresponding miR-485-5p or miR-181c mimics. After transfection for 48 hours, luciferase activity of Firefly and Renilla was determined by a kit of the Dual-Luciferase Reporter Assay System (Promega). Relative luciferase activity of Firefly was measured by normalizing expression ratio to Renilla luciferase activity.

### 2.4. Worldwide Microarray Gene Expression Datasets

Eleven independent Gene Expression Omnibus (GEO) breast cancer microarray datasets (total n=2,248) and two breast cancer datasets (n=2,429) from the Cancer Genome Atlas (TCGA) [24] were collected for this study. All participants had clinical and follow-up annotations. The GEO datasets were GSE7390 [25], GSE2034 [26], GSE1456 [27], GSE4922 [28], GSE22226 [29], GSE24450 [30], GSE53031 [31], GSE25066 [32], GSE10885 [33], GSE58812 [34], and NKI [35]. Datasets without prognostic outcome information were excluded. Detailed information about these downloaded datasets is listed in Suppl.  Table 1. To normalize the mRNA expression levels among all datasets, we restratified all MCAK scores and other related genes into four grades (Q1, Q2, Q3, and Q4) based on the percentile for each dataset. MCAK-low (Q1+Q2) and MCAK-high (Q3+Q4) are also divided by the median value of gene expression.

### 2.5. Gene Set Enrichment Analysis (GSEA)

The GSEA software v3.0 was downloaded from www.broad.mit.edu/gsea and run on the JAVA 8.0 platform [36]. All dataset (.gct) and phenotype label (.cls) files were created and loaded into GSEA software, and gene sets were updated from the above website. The detailed protocol could see our previous publications [37]. Here, the permutations number was 1,000, and the phenotype label was MCAK-high versus MCAK-low.

### 2.6. Data Management and Statistical Methods

After datasets were downloaded from GEO and TCGA websites, the original datasets were converted, merged, and normalized using R 3.4.3 and Python 3.6.3. To make datasets compatible, we prenormalized all participants by Q1, Q2, Q3, and Q4 in each dataset and then merged for pooled analysis. The JMP and R software were used for group comparisons, *χ*2 analysis, Fisher's exact test, and the binomial test of proportions. Kaplan-Meier and Cox models were used to apply for analysis of overall survival (OS) and progression-free survival (PFS). Patients with distant metastasis were excluded in PFS analysis. Multivariate and stratification analyses were applied to reduce the potential confounding effect on the estimation of Hazard Ratio (HR). Missing data were coded and excluded from the analysis.

## 3. Results

### 3.1. MCAK Expression Is Associated with an Aggressive Form of Breast Cancer

**Figure 1:**
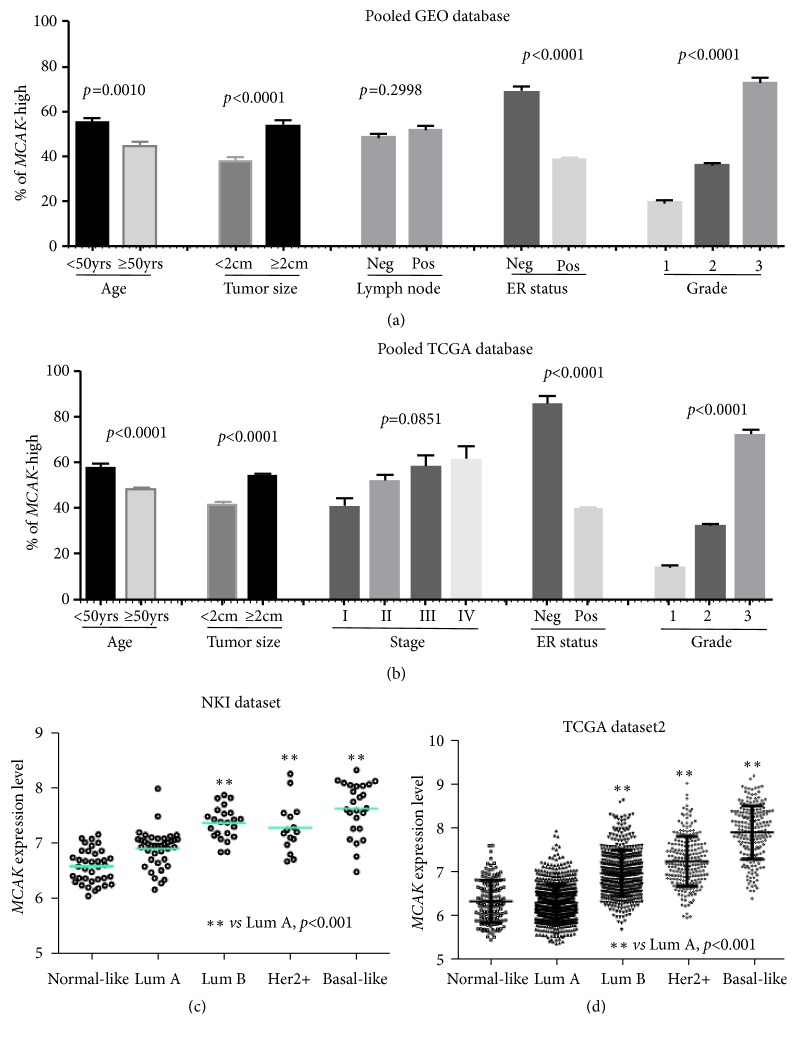
*Clinical relevance of MCAK in GEO and TCGA breast cancer datasets*. Here, MCAK-high was defined as MCAK mRNA level equal to or larger than median mRNA levels in each dataset. The mRNA levels of MCAK, tumor size, lymph node involvement, and Elston grade of breast cancer were analyzed in GEO (a) and TCGA (b) datasets. The mRNA levels of MCAK in different molecular subtypes of breast cancer were also examined in NKI dataset (c) and TCGA dataset (d).

The clinical relevance of MCAK mRNA expression levels was examined on GEO and TCGA datasets. Analysis results from GEO dataset suggest that MCAK expression significantly and positively associated with factors including younger than 50 years of age, tumor equal to or larger than 2 cm, ER-negative status, and higher Elston histology grade (Figure 1(a) and Suppl.  Table 2). However, MCAK expression was not associated with lymph node involvement. These associations from GEO datasets were consistent with findings from the TCGA dataset (Figure 1(b) and Suppl.  Table 2). We further analyzed the MCAK expression on breast cancer patients according to molecular subtypes. ANOVA analysis result confirmed that MCAK mRNA levels were relatively lower on normal-like and Luminal A patients and significantly higher in luminal B, HER2-positive, and basal-like breast cancer cases. This finding was seen in GEO datasets and TCGA datasets (Figures 1(c), and 1(d), and Suppl.  Table 2).

The online search results from the STRING database (https://string-db.org/) [38] indicated that the top 10 proteins that interact with MCAK are the following: Aurora kinase B (AURKB), Baculoviral IAP repeat-containing 5 (BIRC5), Cyclin B1 (CCNB1), Budding uninhibited by benzimidazoles 1 homolog (BUB1), Budding uninhibited by benzimidazoles 1 homolog beta (BUB1B), Cell division cycle 20 (CDC20), Cell division cycle associated 8 (CDCA8), Centromere protein A (CENPA), Centromere protein F (CENPF), and Polo-like kinase 1 (PLK1) (Suppl.  Figure 1). The above proteins are involved in the regulation of mitotic spindle assembly checkpoint, mitotic cell cycle, mitotic nuclear division, and the establishment of chromosome localization. GSEA results indicated that higher expression of MCAK was significantly associated with gene signatures, including Poola invasive breast cancer (up) (Normalized Enrichment Score, NES=1.65, p=0.001) and Riz erythroid differentiation (NES=2.11, p<0.001) (Suppl. Figures 2A and 2B). Meanwhile, MCAK also enriched other cancer invasion related gene sets, such as Mootha mitochondrial, Naderi breast cancer prognosis (up), Biudus metastasis (up), and Zhang breast cancer progenitors (up) (Suppl.  Figure 2C)

Therefore, those above-mentioned large-scale population-based analyses validated that MCAK expression levels were significantly associated with factors related to the aggressiveness of breast cancers.

### 3.2. MCAK Prognosticates Poor Survivability of Breast Cancer

The above findings suggested that MCAK expression was associated with higher Elston grade and other aggressive phenotypes of breast cancer. Here, we hypothesized that the expression of MCAK might be associated with poor outcomes in breast cancer. To address this, we conducted Kaplan-Meier and Cox analysis to determine if MCAK impacted survival in breast cancer cases in GEO and TCGA microarray gene expression datasets. Here, we recategorized participants of each dataset into four subgroups (Q1, Q2, Q3, and Q4) according to the expression levels of MCAK. First, survival analysis was conducted for each dataset by using univariate and multiple Cox proportional hazard analysis (Table 1). The lowest expression subgroup (Q1) was the relative point of reference. The HR of MCAK OS and PFS increased as its expression levels increased in all datasets. In higher MCAK levels (Q4), the significance could be seen in almost all datasets. The adjusted HRs of higher MCAK (Q4) for OS were 2.27 (95% CI 1.30-4.11) and 2.22 (95% CI 1.65-3.01) in pooled GEO and TCGA datasets, respectively.

**Figure 2:**
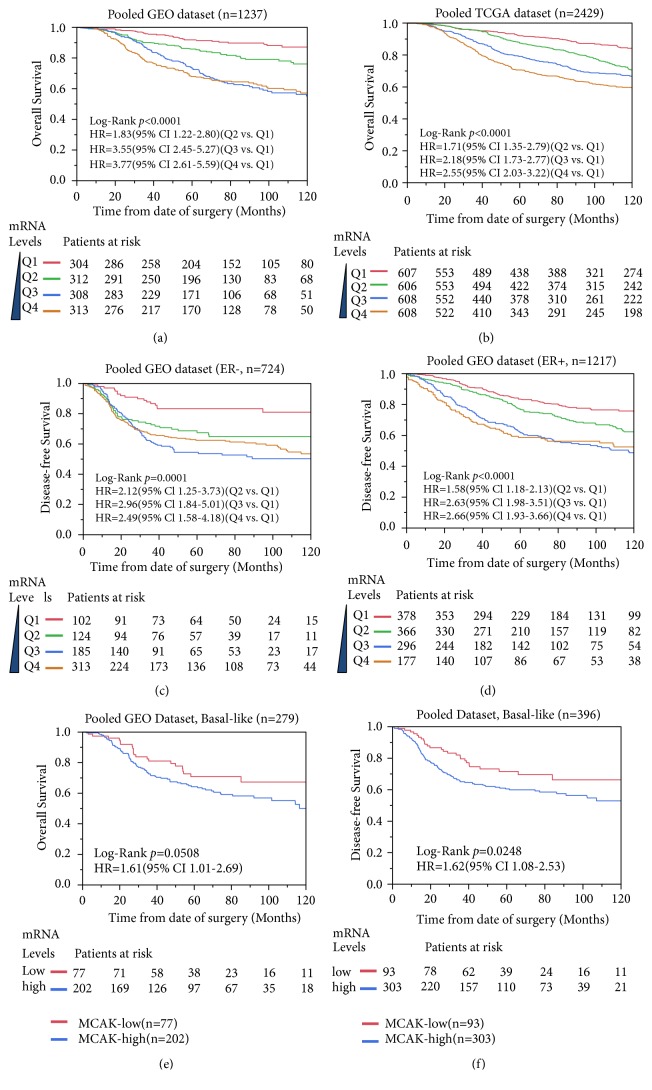
*Survival analysis of MCAK expression in GEO and TCGA breast cancer datasets*. The Kaplan-Meier curves were plotted to visualize MCAK expression levels and outcomes in breast cancer cases. The upper panel listed the overall analysis results of MCAK expression in pooled GEO dataset (a) and TCGA dataset (b). In the middle panel, MCAK was significantly associated with disease-free survival in ER-positive (c) and ER-negative (d) breast cancer patients in pooled GEO datasets. MCAK expression was significantly associated with poor disease-free (e) and overall survival (f) in basal-like breast cancer cases.

The prognostic performance of MCAK was illustrated in Figure 2. The mRNA level of MCAK was significantly associated with poor overall survival in breast cancer on GEO and TCGA datasets (Figures 2(a) and 2(b)). As MCAK levels increased, survival decreased in a dose-dependent manner. Generally, ER-negative breast cancers had a poorer prognosis [39]. We further stratified our Kaplan-Meier analysis and confirmed that MCAK mRNA levels were significantly associated with poor PFS in both ER-negative and ER-positive breast cancers (Figures 2(c) and 2(d)). This finding could also be observed on OS analysis from GEO and TCGA datasets. The prognostic significance of MCAK was also analyzed among molecular subtypes. In the pooled GEO set, MCAK significantly impacted survival in basal-like breast cancer (MCAK-high versus MCAK-low) (Figures 2(e) and 2(f)). Due to insufficient cases of basal-like breast cancers, this association could not be validated in the TCGA dataset. Nevertheless, MCAK prognosticated poor survivability of breast cancer regardless of ER status.

### 3.3. Reduction of MCAK Expression by miR-485-5p and miR-181c on Breast Cancer Cells

**Figure 3:**
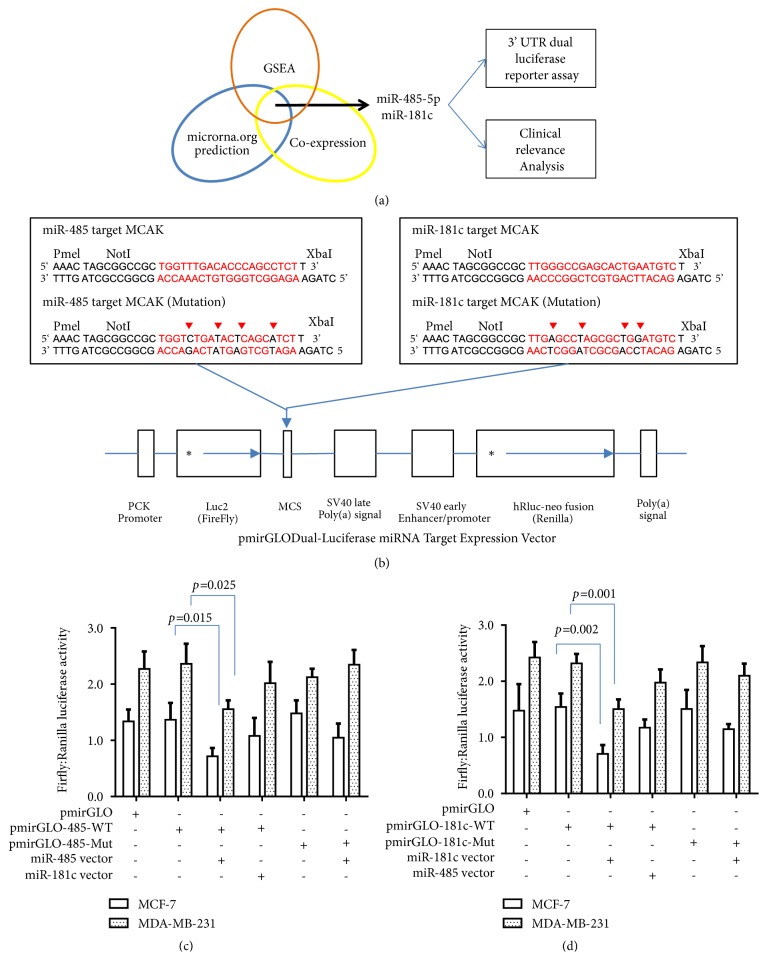
*Identification of microRNAs that modulate expression of MCAK in breast cancer cells*. The strategy to identify microRNA modulating MCAK expression was displayed on (a). First, the prediction of target microRNA for MCAK expression was researched on www.microrna.org. Second, the MCAK enriched microRNA gene signatures were also taken into consideration. Meanwhile, those eligible microRNAs were also significantly and negatively correlated with MCAK mRNA levels. Here, miR-485-5p and miR-181 were selected as eligible microRNAs that target MCAK in breast cancer. The double-strand DNA fragments of MCAK binding sites for miR-485-5p and miR-181c were synthesized (b). Mutation fragments were also synthesized for negative control. For each fragment, the PmeI and XhaI restrict enzyme sequence was inserted, and NotI enzyme sequence also inserted for internal control. These fragments were inserted into multiple cloning sites (MCS) of pmirGLO Dual-Luciferase miRNA Target Expression Vector, which was located on 3' untranslated region (3' UTR) of Firefly luciferase (*luc2)* gene. The pmirGLO-485-5pWT and pmirGLO-181cWT represent wild-type report plasmids of miR-485-5p and miR-181c targeting MCAK, respectively. The pmirGLO-485-5pMut and pmirGLO-181cMut were corresponding to mutants' report plasmids. These report plasmids were transfected into MCF-7 and MDA-MB-231 cells, and luminescence activity was tested after being incubated for 48 hours. The Firefly:Renilla luciferase activity was used to indicate the inhibition rate of reporter systems for miR-485-5p (c) and miR-181c (d), respectively.

In general, microRNAs suppress gene expression level through posttranscriptional regulation. Here, all possible microRNAs that target MCAK were identified based on www.microrna.org website. Meanwhile, the MCAK coexpressing microRNAs were listed from the GSE22220 dataset. GSEA also analyzed the targeting gene sets of microRNA enriched by MCAK. Only those predicted microRNAs, which were also negatively and significantly coexpressed with MCAK, were considered as eligible microRNAs targeting MCAK (Figure 3(a)). Here, two candidate microRNAs, miR-485-5p and miR-181c, were selected. The binding sites and gene map were outlined in Suppl.  Figure 3. A 3'-UTR luciferase reporter assay was used to investigate inhibitory effects of these microRNAs through binding to the corresponding sequence on MCAK. The clinical significance of microRNAs was also evaluated for further validation.

It is based on predicted binding motifs of miR-485-5p and miR-181c that target MCAK mRNA; double-strand DNA fragments were synthesized and inserted into multiple cloning sites (MCS) of pmirGLO Dual-Luciferase miRNA Target Expression Vector (Figure 3(b)). The pmirGLO plasmid was transfected into MCF-7 cells and incubated for 48 hours. The breast cancer cell was harvested and tested by luminescence. The Firefly and Renilla luciferase activity was dramatically higher in pmirGLO-485-5p-WT and pmirGLO-181c-WT transfectants compared to blank control. In Figures 3(c) and 3(d), the analysis indicated that the Firefly and Renilla relative luciferase activities of pmirGLO-485-5p-WT and pmirGLO-181c-WT decreased by more than 50% when they were cotransfected with miR-485-5p and miR-181c expression vectors, respectively (p<0.05). However, the relative luciferase activity of pmirGLO-485-5p-WT was not reduced by the miR-181c mimic. The luciferase activity of pmirGLO-181c-WT was also not inhibited by the miR-485 mimic. On the other hand, miR-485-5p mimic could not quench the luciferase activity of pmirGLO-485-5p-Mut significantly. Similar results also could be seen on pmirGLO-181c-Mut/miR-181c cotransfection. Therefore, this investigation revealed that miR-485-5p and miR-181c would reduce the expression by specifically binding to corresponding motifs of MCAK mRNA.

### 3.4. miR-485-5p and miR-181c Might Suppress MCAK Expression and Associate with Better Outcome in Breast Cancer

**Figure 4:**
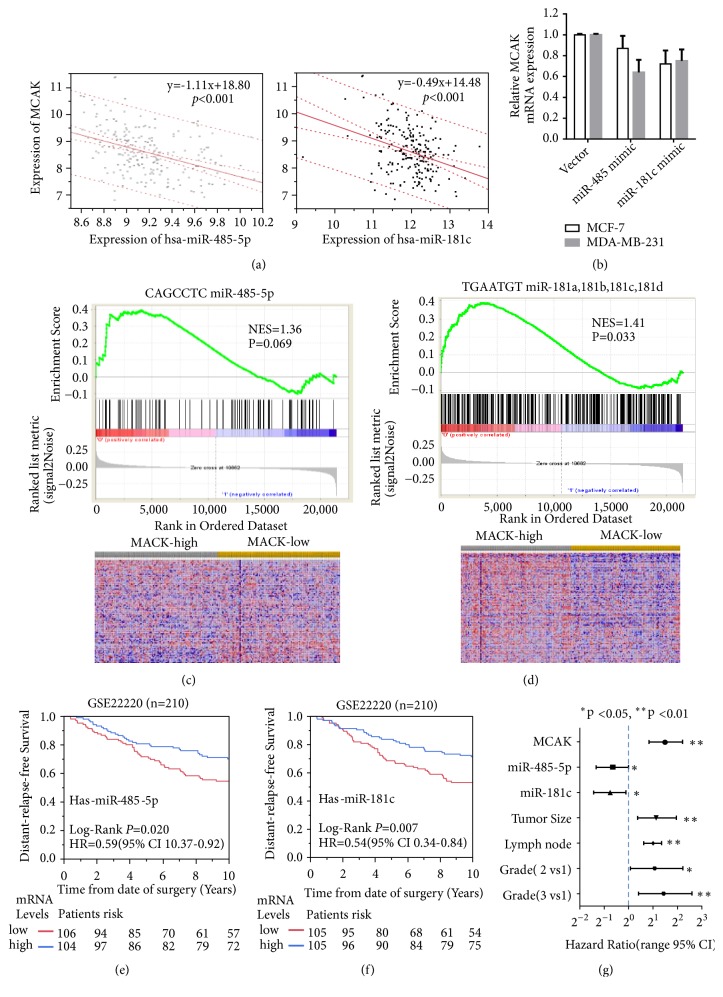
*miR-485-5p and miR-181c negatively correlated with MCAK expression and associated with better survival in breast cancer*. The scatter plots between MCAK and miR-485-5p and miR-181c were shown on (a). The mRNA expression of MCAK was reduced by mimics of miR-485-5p and miR-181c (b). A gene set enrichment analysis for MCAK and signatures of miR-485-5p/miR-181c were also displayed on (c) and (d). Cases were stratified into high and low subgroups based on expression levels of miR-485-5p and miR-181c. The Kaplan-Meier curves of these two microRNAs are shown in (e) and (f). Cox proportional hazard analysis for MCAK, miR-485-5p, miR-181c, tumor size, lymph node involvement, and Elston grade in GSE22220 dataset are shown on (g).

The scatter plot displayed by the expression of MCAK was significantly and negatively correlated with miR-485-5p and miR-181c, respectively (Figure 4(a)) (p<0.001). Meanwhile, the mRNA expression of MCAK in miR-485-5p and miR-181c mimic plasmid transfectants was reduced by 13% and 28%, respectively, in comparison to control vector in MCF-7 cell. Two microRNAs also could suppress MCAK by 36% and 25% in MDA-MB-231 cell (Figure 4(b)). It was reported that miR-485-5p targets PAK1 [14], and miR-181c targets Smad7 [18] and PTEN [19]. Here, the mRNA expressions of PAK1, Smad7, and PTEN were reduced 21%, 12%, and 22% by corresponding mimic plasmids In MCF-7 cell. However, it failed to show statistical significance. Similar results also could be seen in MDA-MB-231 cell. Further, GSEA also demonstrated that MCAK could enrich gene sets of miR-485-5p (CAGCCTC) and miR-181c (TGAATGT) (Figures 4(c) and 4(d)). The NES for miR-485-5p and miR-181c were 1.36 (p=0.069) and 1.41 (p=0.033), respectively.

The clinical relevance of miR-485-5p and miR-181c was analyzed on GSE22220 dataset (Table 2). Here, we stratified breast cancer patients as high and low subgroups based on the median scores of miR-485-5p and miR-181c, respectively. The expression of miR-485-5p and miR-181c was likely associated with age. Interestingly, miR-485-5p was higher in cases of breast patients younger than 50 years (p=0.0632), but miR-181c was significantly higher in 50-year-old or older patients (p=0.0081). Both miR-485-5p and miR-181c were significantly associated with lower Elston histology grade (p values were 0.014 and 0.009, resp.). Also, miR-181c, but not miR-485-5p, was significantly associated with ER-positive status (p=0.0233). Both miR-485-5p and miR-181c were not significantly related to tumor size and lymph node involvement. Because of insufficient clinical data, we could not analyze the clinical relevance of microRNAs on molecular subtypes of breast cancer. Nevertheless, these findings were compatible with previous MCAK clinical relevance data.

A further outcome study was conducted for miR-485-5p and miR-181c in breast cancer databases (Figures 4(e) and 4(f)). Here, Kaplan-Meier analysis visualized both microRNAs were significantly and positively associated with better survival of breast cancers. Further Cox proportional analyses were conducted to compare the prognostic performance of MCAK, miR-485-5p, miR-181c, tumor stage, lymph node stage, and Elston histology grade in breast cancer on GSE22220 dataset (Figure 4(g)). It was shown that MCAK, tumor and lymph node involvement, and histological grade were significantly associated with risk of breast cancer relapse. However, these two microRNAs significantly reduce the relative risk of recurrence (p<0.05). The HRs of miR-485-5p and miR-181c for PFS were 0.59 (95% CI 10.37-0.92) and 0.54 (95% CI 0.34-0.84), respectively. The HR of MCAK was 2.80 (95% CI 1.77-4.57). Therefore, miR-485-5p and miR-181c played opposing roles in MCAK outcome in breast cancer cases.

## 4. Discussion

In this study, analyses were conducted on GEO and TCGA datasets to identify prognostic biomarkers related to MCAK expression in breast cancer. Over 4,600 eligible breast cancer cases were included in this study. Patient profiles composed of multiple ethnicities and social-economic backgrounds (Suppl.  Table 1). Because the gene expression data from each set stems from different platforms and research teams, a key challenge was to integrate all data without any bias systematically. The selection and publication biases were taken into consideration. Individual and pooled analyses were conducted to avoid biases in this study. Also, stratification and multivariate analyses were used to reduce potential confounders. We believe that all findings yielded from this study are repeatable and reliable. Results from individual and pooled analysis consistently revealed that mRNA expression of MCAK was significantly associated with tumor size and Elston histological grade in breast cancer. MCAK expression was also significantly associated with poor outcome of breast cancer in a dose-dependent manner. The analysis results also show that MCAK predicts poor outcome in both ER-positive and ER-negative breast cancers, suggesting that MCAK might promote invasion of breast cancer regardless of ER status. Interestingly, MCAK significantly impacts poor survival in basal-like breast cancer. Even though the clinical relevance and prognostic significance of MCAK protein are not clear, we believe that MCAK might serve as a prognostic biomarker for breast cancer.

The biological mechanism of MCAK involving cancer invasiveness remains unclear. Recent research confirmed that MCAK plays essential roles in depolymerizing microtubules and transporting cargo along microtubules. Moreover, studies have focused on whether MCAK and KIF2A could be induced in mutant K-Ras-transformed cells [40, 41]. Recent studies have found that MCAK regulates lysosomal localization and lysosome organization in immortalized human bronchial epithelial cells (HBECs) [41]. In Ras-transformed cells, MCAK and KIF2A are required for Ras-dependent proliferation and migration to support the transformed phenotype. Depletion of either of these kinesins impairs the ability of cells transformed with mutant K-Ras to migrate and invade Matrigel [40]. However, it seems that depletion of these kinesins could not reverse epithelial to mesenchymal transition (EMT) caused by mutant K-Ras. The mRNA of MCAK dramatically increased in breast cancer tissue in comparison to adjacent normal samples. Inhibition of MCAK with small interfering RNA has inhibited the growth of the breast cancer cell lines T47D and HBC5 [42]. The above findings may explain how overexpression of MCAK plays a critical role in breast carcinogenesis. Nevertheless, further investigation is needed to explore the detailed mechanism of MCAK in cancer proliferation and invasion.

In addition to identifying the association between MCAK and breast cancer aggressiveness, we also demonstrate that microRNAs were related to MCAK. Here, several methodologies confirm that miR-485-5p and miR-181c target MCAK and negatively regulate regulatory steps in cancer development. First, bioinformatic analysis confirmed that miR-485-5p and miR-181c bind to CAGCCTC and TGAATGT motifs in MCAK, respectively (Figure 3(b) and Suppl.  Figure 2). In our study, a dual-luciferase 3'-UTR reporter assay demonstrated that miR-485-5p and miR-181C specifically inhibited Firefly and Renilla relative luciferase actively by 50% by binding to these motifs (Figures 3(c) and 3(d)). Even the mimics of these two microRNAs only suppressed MCAK mRNA expression levels by 13-36% in breast cancer cells, but our population-based analysis also indicated that miR-485-5p and miR-181C are significantly and negatively coexpressed with MCAK in 214 breast cancer cases (p<0.001) (Figures 4(a) and 4(b)). Meanwhile, GSEA also validated that MCAK could enrich gene signatures of CAGCCTC miR-485-5p (NES=1.36, p=0.069) and TGAATGT miR-181a, 181b, 181c, and 181d (NES=1.41, p=0.033), respectively (Figures 4(c) and 4(d)). Previous studies demonstrated that miR-485-5p significantly reduces the invasive ability of breast cancer cells (MCF-7 and MDA-MB-231) [13] and gastric cancer cells (BGC-823 and SGC7901) [17]. Similarly, miR-181c has been included in prognostic signatures related to breast cancer [43, 44]. A study also showed that miR-181c inhibits the migratory and invasive behaviors of SK-N-SH and SH-SY5Y neuroblastoma cells [18]. However, another research team has reported that miR-181c could promote the proliferation and invasive ability in inflammatory breast cancer (SUM149 cells) which accounts for about 6% of breast cancers [19]. Some inconsistent findings might be due to different signaling pathways in cancer development. In our study, all participants included in the pooled analysis are early primary breast cancer patients [45]. Both miR-485-5p and miR-181c play opposing roles on MCAK expression but both are associated with better survival in breast cancer (Figures 4(e) and 4(f)). Overall, our study suggests that miR-485-5p and miR-181c suppress MCAK expression and invasiveness capability of breast cancers by targeting different sites.

## 5. Conclusions

This study demonstrated that mRNA expression of MCAK was significantly associated with poor outcome in breast cancer cases in a dose-dependent manner. Potentially, MCAK can serve as an independent prognostic biomarker for either ER-positive or ER-negative breast cancer. miR-485-5p and miR-181c expressions suppress MCAK gene expression and prognosticate better survival for breast cancer patients.

## Figures and Tables

**Table 1:** Uni- and multivariate analysis for *MCAK* and survival in microarray datasets.

Data set (cases)		Overall survival		Disease-free survival	
		HR (95% CI)	Adjusted HR (95% CI)*∗*	HR (95% CI)	Adjusted HR (95% CI)*∗*
GSE7390					
(n=198)	Q_1_	Reference	Reference	Reference	Reference
	Q_2_	2.93 (1.19-8.23)†	3.14 (1.27-8.84) †	2.30 (1.21-4.62) **†**	2.26 (1.18-4.55) **†**
	Q_3_	4.84 (2.09-13.12) ‡	4.89 (1.96-13.94) ‡	3.41 (1.82-6.76) **‡**	3.67 (1.85-7.62) **‡**
	Q_4_	2.32 (0.90-6.81)	1.72 (0.60-5.75)	1.65 (0.82-3.41)	1.85 (0.80-4.35)
GSE2034					
(n=286)	Q_1_	N/A	N/A	Reference	Reference
	Q_2_			1.56 (0.86-2.89)	1.55 (0.86-2.88)
	Q_3_			2.13 (1.21-3.87) **‡**	2.26 (1.27-4.13) **‡**
	Q4			2.19 (1.24-4.00) **‡**	2.47 (1.35-4.62) **‡**
GSE1456					
(n=159)	Q_1_	Reference	Reference	Reference	Reference
	Q_2_	10.1 (1.90-187) **‡**	1.6e+9	1.8e+9	1.5e+9
			(4.06-2.7e+305) **‡**	(6.03-6.6e+179) **‡**	(4.55-9.0e+304) **‡**
	Q_3_	21.7 (4.46-392) **‡**	3.1e+9	2.9e+9	2.2e+9
			(7.91-3.6e+122) **‡**	(9.71-1.5e+254) **‡**	(6.58-1.8e+34) **‡**
	Q_4_	15.2 (3.03-276) **‡**	1.9e+9	2.5e+9	1.4e+9
			(4.59-6.9e+100) **‡**	(8.38-6.3e+55) **‡**	(3.81-1.2e+137) **‡**
GSE4922					
(n-289)	Q_1_	N/A	N/A	Reference	Reference
	Q_2_			1.25 (0.65-2.41)	1.23 (0.63-2.43)
	Q_3_			1.99 (1.08 -3.76)**†**	1.82 (0.95-3.56)
	Q_4_			2.33 (1.28-4.36) **‡**	1.65 (0.79-3.50)
GSE22226					
(n=129)	Q_1_	Reference	Reference	Reference	Reference
	Q_2_	0.71 (0.14-3.32)	0.92 (0.17-4.99)	0.83 (0.29-2.31)	0.68 (0.20-2.15)
	Q_3_	2.24 (0.70-8.38)	1.73 (0.47-8.30)	1.27 (0.49-3.39)	1.10 (0.39-3.24)
	Q_4_	3.94 (1.37-14.1) **‡**	2.49 (0.71-11.7)	2.95 (1.30-9.27) **‡**	2.18 (0.84-6.15)
GSE24450					
(n=183)	Q_1_	Reference	N/A	Reference	N/A
	Q_2_	0.60 (0.12-2.44)		0.50 (0.10-1.89)	
	Q_3_	2.07 (0.74-6.66)		1.86 (0.69-5.47)	
	Q_4_	4.29 (1.72-12.9) **‡**		4.23 (1.80-11.6) **‡**	
GSE53031					
(n=167)	Q_1_	N/A	N/A	Reference	Reference
	Q_2_			3.58 (1.30-12.6) **†**	2.87 (1.01-10.3) **†**
	Q_3_			2.88 (1.00-10.3) **†**	1.99 (0.66-7.33)
	Q_4_			2.91 (0.99-10.5)	1.30 (0.39-5.16)
GSE25066					
(n=198)	Q_1_	N/A	N/A	Reference	Reference
	Q_2_			2.13 (0.67-7.84)	1.62 (0.49-6.25)
	Q_3_			5.03 (1.86-17.49) **‡**	3.86 (1.33-14.2) **†**
	Q_4_			4.54 (1.66-15.84) **‡**	2.55 (0.80-10.1)
GSE10885					
(n=237)	Q_1_	Reference	Reference	Reference	Reference
	Q_2_	1.79 (0.70-4.89)	1.43 (0.49-4.50)	0.85 (0.35-2.02)	0.95 (0.36-2.44)
	Q_3_	1.75 (0.65-4.91)	1.21 (0.40-3.79)	1.95 (0.94-4.22)	1.75 (0.77-4.17)
	Q_4_	2.68 (1.18-6.85) †	2.01 (0.74-6.13)	1.97 (0.98-4.13)	1.77 (0.75-3.47)
GSE58812					
(n=107)	Q_1_	Reference	Reference	Reference	Reference
	Q_2_	0.73 (0.24-2.10)	0.83 (0.27-2.42)	1.39 (0.48-4.22)	1.54 (0.53-4.70)
	Q_3_	1.13 (0.45-2.96)	1.39 (0.54-3.69)	1.84 (0.70-5.33)	2.22 (1.02-7.63)
	Q_4_	0.55 (0.17-1.66)	0.67 (0.20-2.01)	0.91 (0.29-2.99)	1.10 (0.34-3.55)
NKI set					
(n=295)	Q_1_	Reference	Reference	Reference	Reference
	Q_2_	3.56 (1.28-12.57) †	2.64 (0.92-8.48)	1.87 (0.99-3.70)	1.60 (0.83-3.21)
	Q_3_	9.12 (3.60-30.71) **‡**	5.47 (2.07-18.9) **‡**	3.89 (2.17-7.40) **‡**	2.95 (1.59-5.80) **‡**
	Q_4_	11.16 (4.41-37.54)**‡**	4.39 (1.53-16.0) **‡**	3.95 (2.19-7.55) **‡**	2.37 (1.17-5.00) †
TCGA1					
(n=526)	Q_1_	Reference	Reference	Reference	Reference
	Q_2_	0.68 (0.36-1.27)	0.74 (0.39-1.38)	1.32 (0.63-2.84)	1.39 (0.66-3.07)
	Q_3_	1.01 (0.55-1.84)	1.17 (0.63-2.16)	0.76 (0.30-1.84)	0.79 (0.30-1.96)
	Q_4_	0.94 (0.52-1.70)	1.01 (0.50-2.02)	1.67 (0.83-3.51)	4.25 (0.78-4.25)
TCGA2					
(n=1903)	Q_1_	Reference	Reference	NA	NA
	Q_2_	1.99 (1.53-2.61) **‡**	1.88 (1.43-2.50) **‡**		
	Q_3_	2.50 (1.94-3.26) **‡**	2.08 (1.57-2.78)**‡**		
	Q_4_	3.00 (2.33-3.89)**‡**	2.20 (1.63-2.99) **‡**		
Pooled GEO					
(n=2248)	Q_1_	Reference	Reference	Reference	Reference
	Q_2_	1.83 (1.22-2.80) **‡**	2.04 (1.20-3.60) **‡**	1.64 (1.28-2.11)**‡**	1.54 (1.34-2.09) **‡**
	Q_3_	3.55 (2.45-5.27) **‡**	3.13 (1.87-5.47) **‡**	2.66 (2.11-3.38)**‡**	2.30 (1.71-3.14) **‡**
	Q_4_	3.77 (2.61-5.59)**‡**	2.27 (1.30-4.11) **‡**	2.66 (2.11-3.38)**‡**	1.82 (1.31-2.54)**‡**
Pooled TCGA					
(n=2429)	Q_1_	Reference	Reference	Reference	Reference
	Q_2_	1.71 (1.35-2.19) **‡**	1.85 (1.41-2.45) **‡**	1.32 (0.63-2.84)	1.40 (0.66-3.07)
	Q_3_	2.18 (1.73-2.77) **‡**	2.08 (1.57-2.77) **‡**	0.76 (0.30-1.84)	0.79 (0.30-1.96)
	Q_4_	2.55 (2.03-3.22)**‡**	2.22 (1.65-3.01) **‡**	1.67 (0.83-3.51)	1.79 (0.78-4.25)
					

Note: uni- and multivariate analyses were conducted to evaluate HR of *MCAK*.

*∗*For multivariate analysis, HR was adjusted by age, ER status, and Elston Grade in GSE7390, GSE4922, and GSE25066 and in pool analysis datasets. In the GSE2034 set, HR was adjusted by ER status and it was adjusted by age and ER status in GSE58812. The probe of *MCAK* was 209408_s_at.

HR was adjusted by age, ER status, and Elston Grade in GSE10885 and GSE22226 sets, in which the probe of *MCAK* was A_23_P34788.

The probe of *MCAK* was ILMN_1779153 in GSE24550.

HR was adjusted by age, ER status, and Elston Grade in the GSE53031 set, in which the probe of *MCAK* was 11745868_a_at.

*∗*† Statistical significance, *P*<0.05; ‡ Statistical significance, *P*<0.01.

**Table 2:** Clinical relevance of miR-485 and miR-181c on GSE 22220 dataset.

	has-miR-485-5p			has-miR-181c		
	High (%^*∗*^)	Low	p value†	High (%^*∗*^)	Low	p value†
Age						
<50	41 (58.6)	29		26 (37.1)	44	
≧50	63 (45.0)	77	0.0632	79 (56.4)	61	0.0081
Grade						
1	27 (64.3)	15		26 (61.9)	16	
2	41 (50.0)	41		47 (57.3)	35	
3	40 (35.5)	73	0.0137	22 (35.5)	40	0.0092
Tumor size						
<2cm	38 (58.5)	27		36 (55.4)	29	
>=2cm	66 (45.5)	79	0.0823	69 (47.6)	76	0.2957
Lymph node						
0	59 (49.2)	61		65 (54.2)	55	
1-2	21 (47.7)	23		18 (40.9)	26	
>=3	24 (52.2)	22	0.9084	22 (47.8)	24	0.3036
ER status						
Negative	35 (42.7)	47		33 (40.2)	49	
Positive	69 (53.9)	59	0.1120	72 (56.3)	56	0.0233

Note: there are 1, 1, 1, 5, and 1 missing cases in age, tumor size, lymph node, grade, and ER status.

*∗* % represents positive rate of *has-miR-485-5p/has-miR-181c* is equal to N _High_/(N _High_+N_Low_)×100%.

† *p* values were based on the Pearson Chi-square test.

## Data Availability

The breast cancer datasets supporting this study are available at GEO (https://www.ncbi.nlm.nih.gov/geo/) and TCGA (https://cancergenome.nih.gov) datasets. And the datasets are cited at relevant places within the text as references [6, 8, 9, 15, 24–28, 31, 32, 35, 43, 45].

## Abbreviations

MCAK: Mitotic Centromere-Associated Kinesin, or Kinesin Family Member 2C (KIF2C)
MT: Microtubule
GEO: Gene Expression Omnibus
TCGA: The Cancer Genome Atlas
ER: Estrogen Receptor
PR: Progesterone Receptor
MKI-67: Marker of proliferation Ki-67
HER2: Human Epidermal growth factor Receptor 2
GSEA: Gene Set Enrichment Analysis
OS: Overall survival
PFS: Progression-free survival
HR: Proportional Hazard Ratio
95% CI: 95% Confidence Interval.

## Supplementary Materials

Supplementary Materials Supplementary Table 1: summary of worldwide breast cancer gene expression datasets. Supplementary Table 2: expression of MCAK/Kif2C and clinical features of breast cancer. Supplementary Figure 1: MCAK/KIF2C protein interaction network. Supplementary Figure 2: gene set enrichment analysis (GSEA) for MCAK enriched gene signatures. Supplementary Figure 3: the binding location and binding patterns of microRNAs on MCAK gene.
