# Supplementary material for: Human Mitotic Centromere-Associated Kinesin Is Targeted by MicroRNA 485-5p/181c and Prognosticates Poor Survivability of Breast Cancer
Source: J Oncol. 2019 Apr 3;2019:2316237. doi: 10.1155/2019/2316237 (PMC6470426; doi:10.1155/2019/2316237)
Supplement: Supplementary Materials — Supplementary Table 1: summary of worldwide breast cancer gene expression datasets. Supplementary Table 2: expression of MCAK/Kif2C and clinical features of breast cancer. Supplementary Figure 1: MCAK/KIF2C protein interaction network. Supplementary Figure 2: gene set enrichment analysis (GSEA) for MCAK enriched gene signatures. Supplementary Figure 3: the binding location and binding patterns of microRNAs on MCAK gene. [file 2316237.f1.docx]

**Supplementary materials**

We collected eleven independent Gene Expression Omnibus (GEO) breast cancer microarray datasets and two breast cancer datasets from The Cancer Genome Atlas (TCGA) for this study. Detailed information was listed in Suppl.Table1. Besides, we analyzed the MCAK mRNA expression in GEO and TCGA datasets and examined its clinical relevance in Suppl.Table2. Analysis results suggest that MCAK expression significantly and positively associated with factors including younger than 50 years of age, tumor equal or larger than 2 cm, ER-negative status, and higher Elston histology grade. Meanwhile, MCAK mRNA levels were relatively lower on normal-like, and Luminal A patients, and significantly higher in luminal B, HER2-positive, and basal-like breast cancer cases.

The online search results from the STRING database (https://string-db.org/) indicated that the top 10 proteins that interact with MCAK were shown in Suppl. Fig 1. GSEA results indicated that higher expression of MCAK was significantly associated with gene signatures, including Poola invasive breast cancer (up) (Normalized Enrichment Score, NES=1.65, p=0.001) and Riz erythroid differentiation (NES=2.11, p<0.001) (Suppl. Fig. 2A and 2B).Meanwhile, MCAK also enriched other cancer invasion related gene sets, such as:

209 Mootha mitochondrial, Naderi breast cancer prognosis (up), Biudus metastasis (up), and

210 Zhang breast cancer progenitors (up) (Suppl. Fig. 2C).

We identified microRNA that modulates MCAK expression through three steps. First, the prediction microRNAs for targeting MCAK expression was researched on [www.microrna.org](http://www.microrna.org). Second, the MCAK enriched microRNA gene signatures were also taken into consideration. Meanwhile, those eligible microRNAs were also significantly and negatively correlated with MCAK mRNA levels. Here, miR-485-5p and miR-181 were selected as eligible microRNAs that target MCAK in breast cancer. The binding sites and gene map were outlined in Suppl.Fig.3.

**Supplementary Table 1. Summary of world-wide breast cancer gene expression datasets**

| Accession No. | Assessable  cases | Date of study | Platforms* | Country | Age at diagnosis | OS  months | PFS  months |
| --- | --- | --- | --- | --- | --- | --- | --- |
| GSE7390 | 198 | 1980-1998 | GPL96 | Canada | 46(24-60) | 4.9-303.6 | 4-231.4 |
| GSE2034 | 286 | 1980-1995 | GPL96 | USA | N/A | N/A | 2.0-171.0 |
| GSE1456 | 159 | 1994-1996 | GPL96, GPL97 | Sweden | N/A | 2.2-101.9 | 2.2-101.9 |
| GSE4922 | 289 | 1987-1989 | GPL96, GPL97 | Singapore | 63(28-93) | N/A | 0-153.0 |
| GSE22226 | 129 | N/A | GPL1708, GPL4133 | USA | 48(31-65) | 9.5-82 | 6.0-84.0 |
| GSE24450 | 183 | 1997-2004 | GPL6947 | Finland | 44(26-53) | 22.4-120 | 0.8-60 |
| GSE53031 | 167 | 1996-2010 | GPL13667 | Belgium | 36(29-47) | N/A | 2.0-156.0 |
| GSE25066 | 198 | N/A | GLP96 | USA | 49(24-72) | N/A | 1.7-88.3 |
| GSE10885 | 237 | N/A | GPL885, GPL887 | USA | 55(24-88) | 1-96 | 1-96 |
| GSE58812 | 107 | 1998-2007 | GPL570 | France | 57(28-84) | 1-171.7 | 1-171.7 |
| NKI# | 295 | 1984-1995 | Agilent 25K Chip | Netherlands | 44(26-53) | 1.0-220.1 | 1.0-220.1 |
| TCGA Dataset-1 | 526 | N/A | N/A | N/A | 58(26-90) | 0-282.7 | 0-281 |
| TCGA Dataset-2 | 1094 | N/A | N/A | N/A | 62(22-96) | 0-355 | N/A |

* Platforms: GPL96: [HG-U133A] Affymetrix Human Genome U133A Array; GPL97: [HG-U133B] Affymetrix Human Genome U133B Array; GPL1708: Agilent-012391 Whole Human Genome Oligo Microarray G4112A ; GPL4133: Agilent-014850 Whole Human Genome Microarray 4x44K G4112F; GPL6947: Illumina HumanHT-12 V3.0 expression beadchip; GPL13667: [HG-U219] Affymetrix Human Genome U219 Array; GPL885: Agilent-011521 Human 1A Microarray G4110A; GPL887: Agilent-012097 Human 1A Microarray (V2) G4110B; GPL570: [HG-U133_Plus_2] Affymetrix Human Genome U133 Plus 2.0 Array.

**Supplementary Table 2. Expression of MCAK/Kif2C and clinical features of breast cancer**

|  | **Pooled GEO dataset** | | | **TCGA dataset** | | |
| --- | --- | --- | --- | --- | --- | --- |
|  | **High(%^*^)** | **Low(%^*^)** | ***p* value**† | **High(%^*^)** | **Low(%^*^)** | ***p* value**† |
| **Age** |  |  |  |  |  |  |
| <50 yrs | 466(53.1) | 411(46.9) |  | 320(57.4) | 238(42.7) |  |
| ≥50 yrs | 391(44.6) | 466(53.4) | 0.001 | 896(47.9) | 976(52.1) | <0.001 |
| **Elston Grade** |  |  |  |  |  |  |
| 1=Well | 47(19.6) | 193(80.4) |  | 23(13.9) | 142(86.1) |  |
| 2=Mod | 231(35.9) | 412(64.1) |  | 237(32.0) | 504(68.0) |  |
| 3=Poor | 496(72.3) | 190(27.7) | <0.001 | 666(71.9) | 260(28.1) | <0.001 |
| **ER** |  |  |  |  |  |  |
| Negative | 504(68.7) | 230(31.3) |  | 466(85.4) | 80(14.6) |  |
| Positive | 474(38.7) | 750(61.3) | <0.001 | 732(39.6) | 1115(60.4) | <0.001 |
| **Tumor size** |  |  |  |  |  |  |
| <2cm | 107(37.7) | 177(62.3) |  | 245(41.3) | 348(58.7) |  |
| ≥2cm | 339(53.7) | 292(46.3) | <0.001 | 695(53.8) | 598(46.3) | <0.001 |
| **Lymph node status** |  |  |  |  |  |  |
| Negative | 456(48.4) | 487(51.7) |  | 125(49.0) | 130(51.0) |  |
| Positive | 236(51.3) | 224(48.8) | 0.300 | 136(52.3) | 124(47.7) | 0.456 |
| **Molecular subtype** |  |  |  |  |  |  |
| Normal-like | 20(12.8) | 136(87.2) |  | 36(25.7) | 104(74.3) |  |
| Luminal A | 56(18.8) | 242(81.2) |  | 116(17.1) | 563(82.9) |  |
| Luminal B | 125(55.1) | 102(44.9) |  | 308(66.8) | 153(33.2) |  |
| HER2 + | 101(68.2) | 47(31.8) |  | 184(83.6) | 36(16.4) |  |
| Basal-like TNBC | 324(77.3) | 95(22.7) | <0.001 | 193(97.0) | 6(3.0) | <0.001 |

Note: In GEO dataset, there are 1529, 1569, 1958, 915, 1403 and 1272 cases in Age, Grade, ER, Tumor size, Lymph node status, and Molecular subtype. While in TCGA dataset, There are 2430, 1832,1886,2393, 2422,2276,1699,515, 506, 1927 ,1904,1904and 1904cases in Age, Elston histological grade, tumor size, ER status, PR status , Her2 status, molecular type ,lymph node status, chemotherapy adjuvant.

* % represent the positive rate of *Kif2c*-High it equal to N _High_/(N _High_+N_Low_)×100%.

† *p* values were based on the Pearson Chi-square test.

**
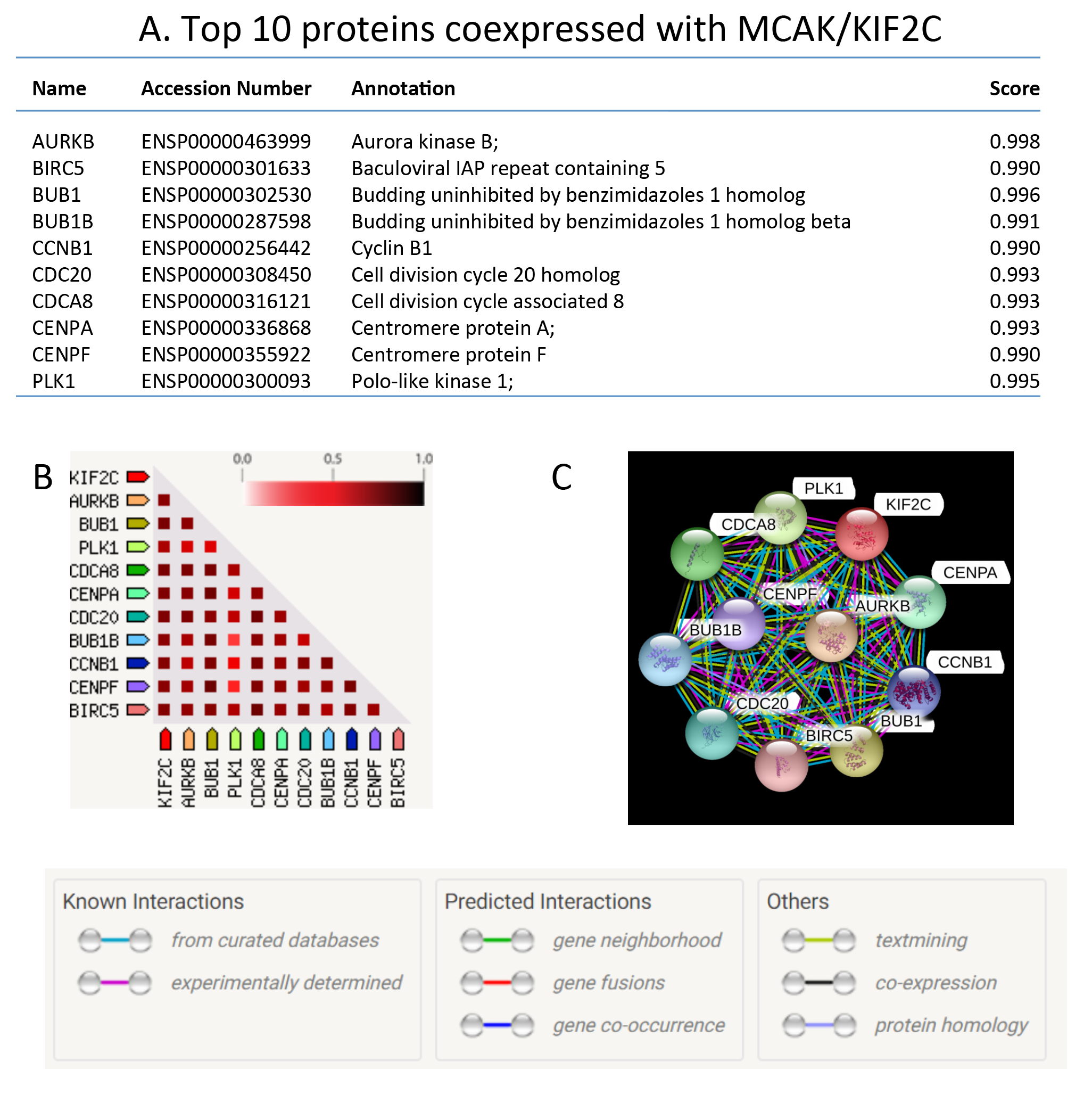
**

**Supplementary Figure 1. MCAK/KIF2C protein interaction network.**

The proteins interacting with MCAK/KIF2C are observed on the STRING database (<https://string-db.org/>). The top 10 MCAK interacting proteins are shown in (A). The interaction between these proteins was shown on (B). Meanwhile, (C) shows the interactions of each other, and the lines with different color represent different intentions.

**
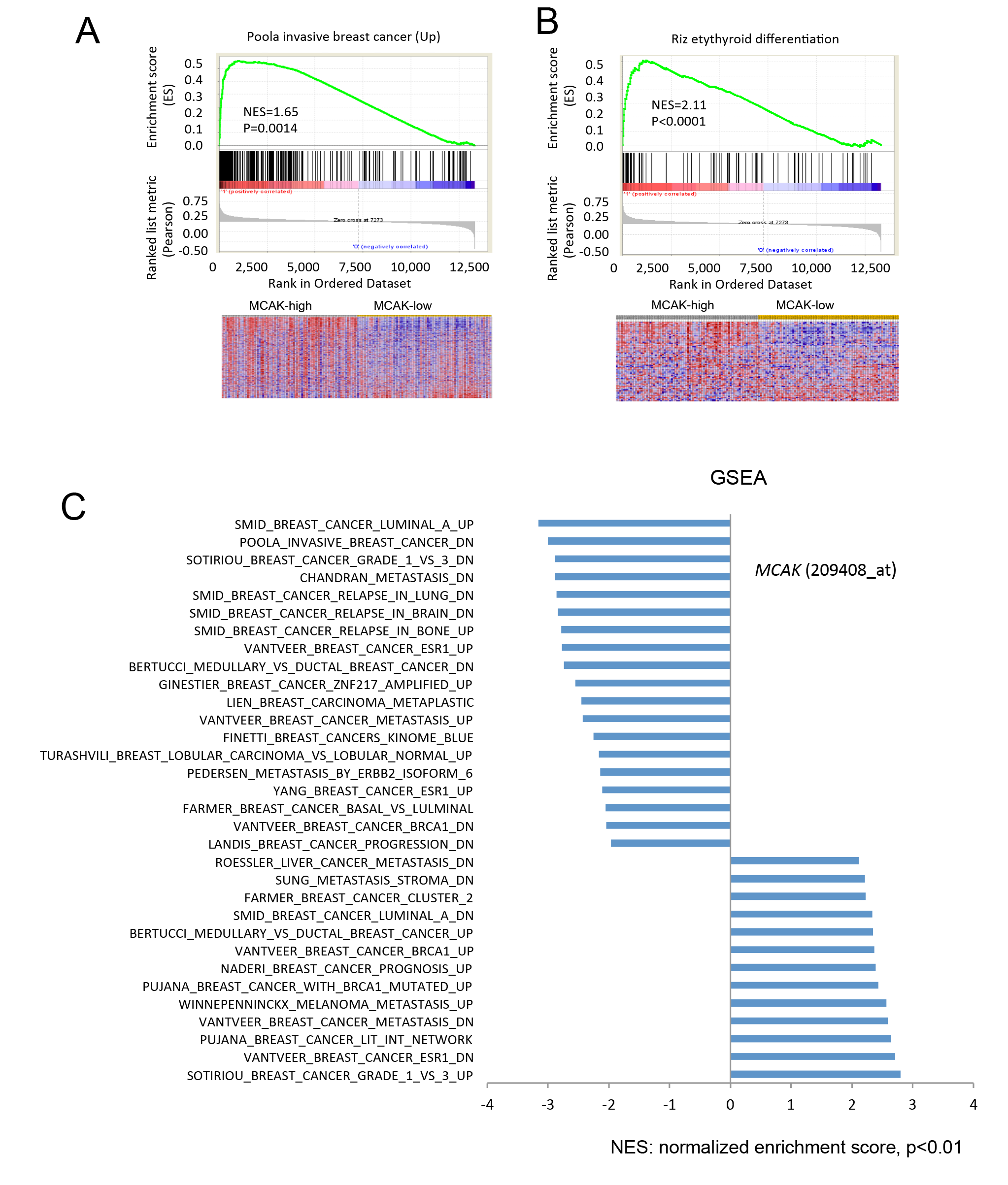
**

**Supplementary Figure 2.** **Gene set enrichment analysis (GSEA) for MCAK enriched gene signatures.**

GSEA was employed to investigate MCAK association with gene signatures related to cancer development. NES represents normalized enrichment score. In heatmap, MCAK-high subgroup was shown on the left, and MCAK-low was shown on the right. Rows list the genes of each signature. The red shows up-regulation, and blue shows down-regulation. Detailed GSEA results of Poola invasive breast cancer (up) gene signature (NES=1.65, p=0.001) and Riz erythroid differentiation (NES=2.11, p<0.001) are shown on (A) and (B). Other cancer invasion related gene signatures enriched by MCAK are listed on (C).


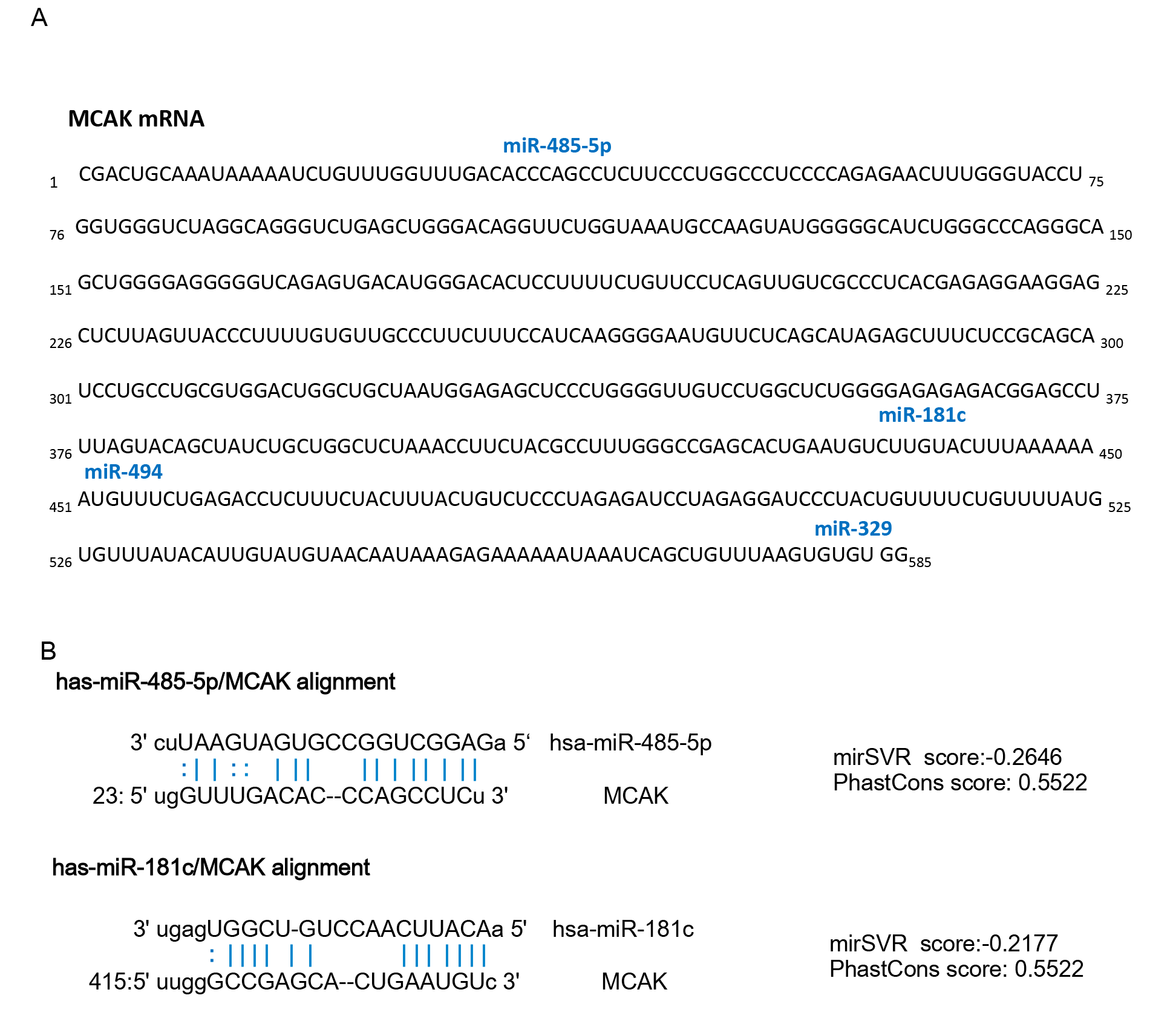


**Supplementary Figure 3. The binding location and binding patterns of microRNAs on MCAK gene.**

Four microRNAs have been predicted having binding sites in MCAK mRNA sequence, as well as coexpressing with MCAK egatively. These microRNAs include miR-485-5p, miR-181c, miR-494, and miR-329（A）. Here, miR-485-5p and miR-181 are selected as eligible microRNAs because GSAE results further suggested that overexpression of MCAK could enrich the gene signature of these two microRNAs. The double-stranded DNA fragments of MCAK binding sites for miR-485-5p and miR-181c were displayed on (B).
